# Your period and your pregnancy, a cohort study of pregnant patients investigating the associations between menstruation and birth outcomes in Australia: study protocol

**DOI:** 10.1136/bmjopen-2024-091813

**Published:** 2025-01-22

**Authors:** Kirstin Tindal, Fiona Cousins, Kirsten Rebecca Palmer, Stacey Ellery, Beverley Vollenhoven, Caroline E Gargett, Adrienne Gordon, Billie Bradford, Miranda Davies-Tuck

**Affiliations:** 1 The Ritchie Centre at Hudson Institute of Medical Research, Clayton, Victoria, Australia; 2 Obstetrics and Gynaecology, Monash University School of Clinical Sciences at Monash Health, Clayton, Victoria, Australia; 3 Women's Health Research Program, Monash Health, Melbourne, Victoria, Australia; 4 Department of Paediatrics, University of Sydney - Camden Campus, Camden, New South Wales, Australia

**Keywords:** Pregnancy, Fetal medicine, OBSTETRICS, GYNAECOLOGY, Dysmenorrhea, PUBLIC HEALTH

## Abstract

**Abstract:**

**Introduction:**

Early pregnancy care involves the screening and identification of women with risk factors for adverse pregnancy outcomes, including stillbirth or preterm birth, to tailor pregnancy care and interventions accordingly. Most stillbirths and approximately two-thirds of preterm births, however, occur in the absence of evident risk factors. The majority of stillbirths occur in the preterm period, yet there are few interventions targeting this period, and progress to reduce stillbirth rates remains slow. Placental dysfunction is a major contributor to stillbirth, particularly, preterm stillbirth. Here, the endometrial environment may shed light on factors that influence placental development and the trajectory of a pregnancy. Menstrual symptoms or abnormal uterine bleeding (AUB) can indicate endometrial disorders, which are associated with infertility and adverse pregnancy outcomes. Whether AUB is associated with pregnancy outcomes in the absence of a diagnosed endometrial pathology, however, remains unknown. Limited information regarding a woman’s menstrual cycle is captured in routine early pregnancy assessments, such as the last menstrual period and menstrual cycle length. Given the latent diagnosis of endometrial disorders and that up to a third of all women experience AUB during their lifetime, determining the association between menstrual characteristics and pregnancy outcomes has the potential to uncover new clinical strategies to reduce adverse pregnancy outcomes. Therefore, this study aims to understand the association between menstruation and pregnancy outcomes to identify which menstrual characteristics could provide value as a pregnancy risk assessment tool.

**Methods and analysis:**

This is a prospective study of women aged 18–45 with a singleton pregnancy. Participants will be recruited in early pregnancy at their antenatal appointment and not have a known diagnosed endometrial pathology (endometriosis, adenomyosis, endometrial cancer or an endometrial submucosal fibroid) or have had an endometrial ablation. Participants will also be excluded if there is a planned termination of pregnancy or a termination of pregnancy for psychosocial reasons. Women will complete a menstrual history survey to capture menstrual cycle length, regularity, level of pain, heaviness of flow and other menstrual symptoms. Participants will consent to having the survey data linked with their pregnancy and birth outcome information. The primary outcome is a composite of stillbirth, spontaneous preterm birth, pre-eclampsia or fetal growth restriction. Participants will also be invited to complete an optional fetal movements survey at 28–32 and 36+ weeks’ gestation, and consent for placental collection at the time of birth will be sought.

**Ethics and dissemination:**

Ethics approval was obtained from Monash Health Human Research Ethics Committee (83559) on 24 April 2024. The study will be conducted in accordance with these conditions. Findings will be disseminated through peer-reviewed publications and conference presentations.

**STRENGTHS AND LIMITATIONS OF THIS STUDY**

- This study will capture responses to a validated menstrual survey early in pregnancy and be prospectively linked to birth outcomes using routinely collected data, minimising measurement bias.
- Women will be recruited to complete a survey while in the waiting room of the antenatal clinic, with no further obligation to the participant, limiting loss to follow-up.
- Participants could have underlying, undiagnosed endometrial pathologies.
- Menstrual characteristics may differ between primigravida and multiparous women, particularly, if there are limited menstrual cycles between pregnancies.

## Introduction

 In recent years, the recognition and importance of reducing stillbirth have increased, having historically been neglected.^1 2^ Interventions such as the ‘Safer Baby Bundle’ in Australia and the ‘Saving Babies Lives’ care bundle in the UK target key risk factors for stillbirth and have been successfully implemented.^3 4^ Despite the promise of such strategies, they target later gestations; however, most stillbirths in Australia occur in the extremely preterm period, between 20 and 28 weeks’ gestation.^5^ There are limited clinical interventions of mixed efficacy to prevent preterm stillbirth and several knowledge gaps we must address to inform future directions in stillbirth prevention. Spontaneous preterm birth, fetal growth restriction (FGR) and antepartum haemorrhage combined account for over a quarter of preterm stillbirths in Australia and an additional 15% of preterm stillbirths are classified as unexplained.^5 6^ Better understanding, and investigation of unexplained stillbirths and the causes of preterm stillbirth are vital to address stillbirth rates overall.

Placental dysfunction is a major contributor to stillbirth^7^, however, the origin of placental dysfunction remains unknown. There is now growing evidence that the endometrial environment in which the embryo implants impacts not only the establishment of pregnancy and placental development^8^,^13^ but is also associated with several adverse pregnancy outcomes.^13^,^16^ Throughout the menstrual cycle, the endometrium undergoes critical structural changes, including the decidualisation of endometrial stromal cells, to support the invasive implantation of a blastocyst and subsequent placentation. In the absence of a pregnancy, decidualised cells are shed during menstruation to start the cycle anew. Abnormalities in endometrial decidualisation are associated with adverse pregnancy outcomes, including recurrent pregnancy loss, pre-eclampsia and preterm birth.^13^,^16^

There is evidence of endometrial dysfunction underpinning adverse pregnancy outcomes such as pre-eclampsia and preterm birth. Endometrial stromal cells from women who previously experienced severe pre-eclampsia have dysfunctional cytoskeletal reorganisation when triggered to decidualise and secrete lower concentrations of prolactin (PRL) and insulin-like growth factor-binding protein 1 (IGFBP-1) compared with controls.^13^ Additionally, global transcriptional profiles of women who had experienced pre-eclampsia were altered (15 genes downregulated and seven upregulated) compared with controls after decidualisation.^13^ The identified genes include those coding for proteins involved in oxygen metabolism, insulin secretion, proliferation, cytokine-receptor interactions and inflammation.^13^ Similar findings are reported in placental chorionic villous samples from women who developed pre-eclampsia, showing altered expression of decidualisation markers, PRL and IGFBP-1, compared with controls,^17 18^ which may be due to decidual senescence before pregnancy. Abnormalities in the decidua and genes that regulate decidual senescence, including cell growth, metabolism and inflammation, have also been associated with spontaneous preterm birth.^15^ So far, no studies have examined the endometrial contribution to FGR and stillbirth in this way. Still, given the common pathways to these adverse outcomes, there will likely be impaired decidualisation.

Currently, clinical investigation of the endometrium is performed via invasive endometrial biopsies and only when there is an indication to do so, such as infertility investigations or for suspected endometriosis. We have recently demonstrated that menstrual fluid, collected non-invasively using a menstrual cup, contains endometrial tissue of comparable composition to late-secretory stage biopsy-derived tissue^19^ and has immense potential as a diagnostic tool.^20 21^ Given that only half of women planning a pregnancy access preconception care^22^ and menstrual fluid cannot be collected from pregnant patients, this tool cannot be used in pregnancy. Understanding menstrual history, however, may overcome this challenge. Self-reported menstrual characteristics, including irregular menstrual cycles and early onset of menarche (first menstruation before 12 years old), are associated with an increased risk of pre-eclampsia,^23^,^25^ FGR^23^ and preterm birth.^26^ Other menstrual symptoms, such as heavy menstrual bleeding, defined as excessive menstrual blood loss (>80 mL) that interferes with a woman’s physical, emotional, social and quality of life, and dysmenorrhea (severe period pain) are common, with each impacting at least 1 in 4 women^27^,^29^. The overall relationship between abnormal uterine bleeding (AUB) and pregnancy outcomes, however, has yet to be explored. Many of these symptoms are also indicative of an endometrial pathology, such as endometriosis; however, diagnosis for endometriosis is complex, often taking up to a decade.^30^ Considering this latent diagnosis, the overlapping risk factors and impaired decidualisation properties between endometrial pathologies and adverse pregnancy outcomes,^30^ it is vital to understand if AUB places women at elevated risk of adverse pregnancy outcomes. To our knowledge, this has not been comprehensively explored.

Menstrual health is indicative of endometrial health, and a better understanding of menstrual characteristics can provide a window into the endometrial environment. The aims of this study are, therefore, to undertake a prospective cohort study to quantify the association between self-reported characteristics of a woman’s menstrual cycle (age of menarche, length and regularity, heaviness of flow, symptoms, and levels of pain) in the 3–6 months before becoming pregnant with maternal, obstetric and perinatal outcomes, and macroscopic and microscopic differences in placental pathology. We hypothesise that even in the absence of a diagnosed endometrial pathology, components of the menstrual history will be associated with adverse pregnancy and perinatal outcomes. This study will uncover previously unknown associations between menstruation and pregnancy and the outcomes have the potential to change pregnancy risk screening and clinical care. They will also open new directions in pregnancy research, elucidating the foundation of placental dysfunction or unknown causes of stillbirth and help drive the development of new interventions and strategies to reduce adverse pregnancy outcomes.

## Methods and analysis

### Study design

This is a prospective cohort study of pregnant women presenting for antenatal care with a singleton pregnancy. The study will be conducted and reported according to the Strengthening the Reporting of Observational Studies in Epidemiology (STROBE) guidelines for cohort studies.^31^

### Study setting

A large, publicly funded health service in Victoria, Australia, Monash Health provides care for ~12 000 women per annum of all acuity levels across four hospitals. Recruitment will be undertaken in 2024 and 2025, with data collection occurring throughout pregnancy.

### Participants

Women aged 18–45 years who are currently pregnant with a singleton fetus and intending to give birth at Monash Health will be invited to participate. Women will be excluded from participating if there is a planned termination of pregnancy, a termination of pregnancy for psychosocial reasons, they have had an endometrial ablation, or they have a clinically diagnosed endometrial pathology (eg, endometriosis, adenomyosis, endometrial cancer or an endometrial submucosal fibroid).

### Recruitment and study procedures

**Figure 1:**
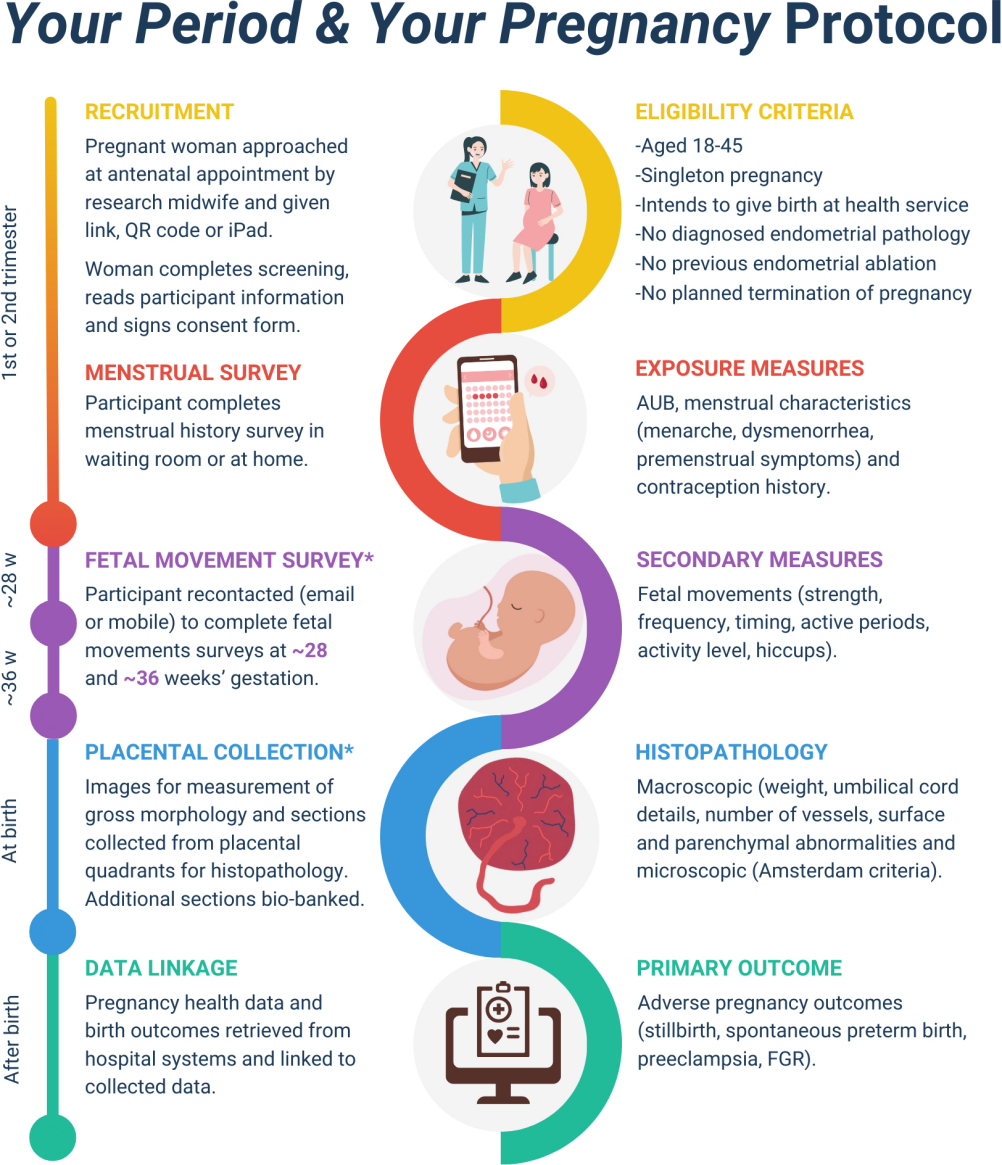
Your period and your pregnancy study protocol. An asterisk (*) denotes optional components of the study.

See figure 1 for an overview of the recruitment procedure. Participants will be approached in the waiting rooms of the antenatal clinic when attending a face-to-face antenatal appointment and invited to participate. At this time, women will have the study aims explained to them, and if they are interested in participating, they will be provided with a link, QR code or study iPad to access a menstrual history survey. Participants will provide written digital consent to have their maternity care records accessed for the purpose of the study and have the option to download or email the participant information and consent form to themselves. Print materials (ie, posters) with QR codes and recruitment information for the study will also be displayed in the waiting room of the antenatal clinic, and digital advertisements may also be used in the telehealth waiting room. Translated versions of recruitment materials and the survey will also be available for non-English-speaking women in the health service’s top four languages (Dari, Vietnamese, Khmer, Mandarin).

Participants who do not complete the survey while at the clinic will be sent up to two automatic reminders to complete the survey. Data will be quality-checked at regular intervals of the study to ensure completion. Written consent will also be sought to contact participants with an optional fetal movement survey later in pregnancy and for placental collection and biobanking. We do not anticipate significant loss to follow-up as all data will be extracted from routinely collected pregnancy information. A research midwife will check hospital systems weekly to identify women who have experienced a pregnancy loss or preterm birth to ensure they are not sent the fetal movement surveys.

Alerts will be created in the hospital system for when participants who have consented to placental collection present to the birthing suite. After the birth, the placenta will be weighed and photographed (maternal surface, fetal surface and cord). Then, a full-depth placental tissue biopsy will be taken before sending the remaining placenta to histopathology (if clinically indicated). Sections of placental tissue will then be bio-banked before being assessed by a blinded clinical pathologist and for future placental scientific examination.^32^

### Variables

#### Primary outcome

Adverse pregnancy outcome: A composite of stillbirth (fetal death before the birth of a baby of 20 or more completed weeks’ gestation or with a birth weight of 400 g or more),^33^ spontaneous preterm birth (birth before 37 weeks of pregnancy), pre-eclampsia (as per ISSHP classification)^34^ and FGR (<3rd centile at birth or diagnosed as per Delphi consensus classification).^35^

#### Secondary outcomes

- Individual components of the primary outcome.
- Macroscopic placental histopathology (eg, placental weight, site of umbilical cord insertion, umbilical cord length, umbilical cord coiling or other cord abnormalities, number of vessels in the cord, maternal and fetal surface abnormalities, and parenchymal abnormalities).
- Microscopic placental findings, grouped according to Amsterdam criteria^36^ (maternal vascular malperfusion, fetal vascular malperfusion, delayed villous maturation, patterns of ascending intrauterine infection and villitis of unknown aetiology as previously described).^37^
- Neonatal death (death of a live born within 7 days after birth).^6^
- Gestation at birth (weeks).
- Indices of fetal growth (baby birth weight (grams), birth weight centile,^38^ growth velocity determined by serial symphysial fundal height (cm) measurements).
- Documented obstetric complications (gestational diabetes, gestational hypertension, preterm prelabour rupture of membranes, bleeding in early pregnancy, hyperemesis, gestational hypothyroidism, gestational thrombocytopaenia, abnormal placentation (antepartum haemorrhage, abruption, previa, accreta, percreta)).
- Presentations for fetal movements (number).
- Findings and actions from clinical investigations and tests (full blood examination, ferritin, thyroid-stimulating hormone, Von Willebrand Factor, 20-week ultrasound (if performed and if significant anomaly found), aspirin prescribed (yes/no), antenatal assessments of fetal well-being (amniotic fluid index, estimated fetal weight, cardiotocography (CTG) and if monitoring triggered iatrogenic birth (yes/no))).
- Labour onset and indication for iatrogenic birth, if relevant.
- Method of birth and indication for operative birth, if relevant.
- Intrapartum compromise (eg, fetal decelerations, bradycardia, abnormal CTG).
- Maternal blood loss (mls), maternal blood transfusion, if relevant.
- Apgar score <7 at 5 min^39^ and need for resuscitation.
- Admission to the neonatal intensive care unit or special care nursery and indication.
- Cause of stillbirth or neonatal death (if applicable) as per PSANZ criteria^40^ (including timing).
- Maternal self-report of fetal movements (strength, frequency, movement timing, active periods, activity level, hiccups) between 28 and 36 weeks’ gestation (if survey completed).

### Exposure measures

#### Primary

AUB is defined as one or more of the following^28^:

- Heavy menstrual bleeding (heavy (patient determined) or prolonged (>8 days)).
- Metrorrhagia (intermenstrual bleeding).
- Oligomenorrhea (irregular menstrual bleeding, cycle variation ≥8 days).
- Hypomenorrhea (light bleeding, patient determined).
- Amenorrhoea (absence of menstruation).

#### Secondary

**Figure 2:**
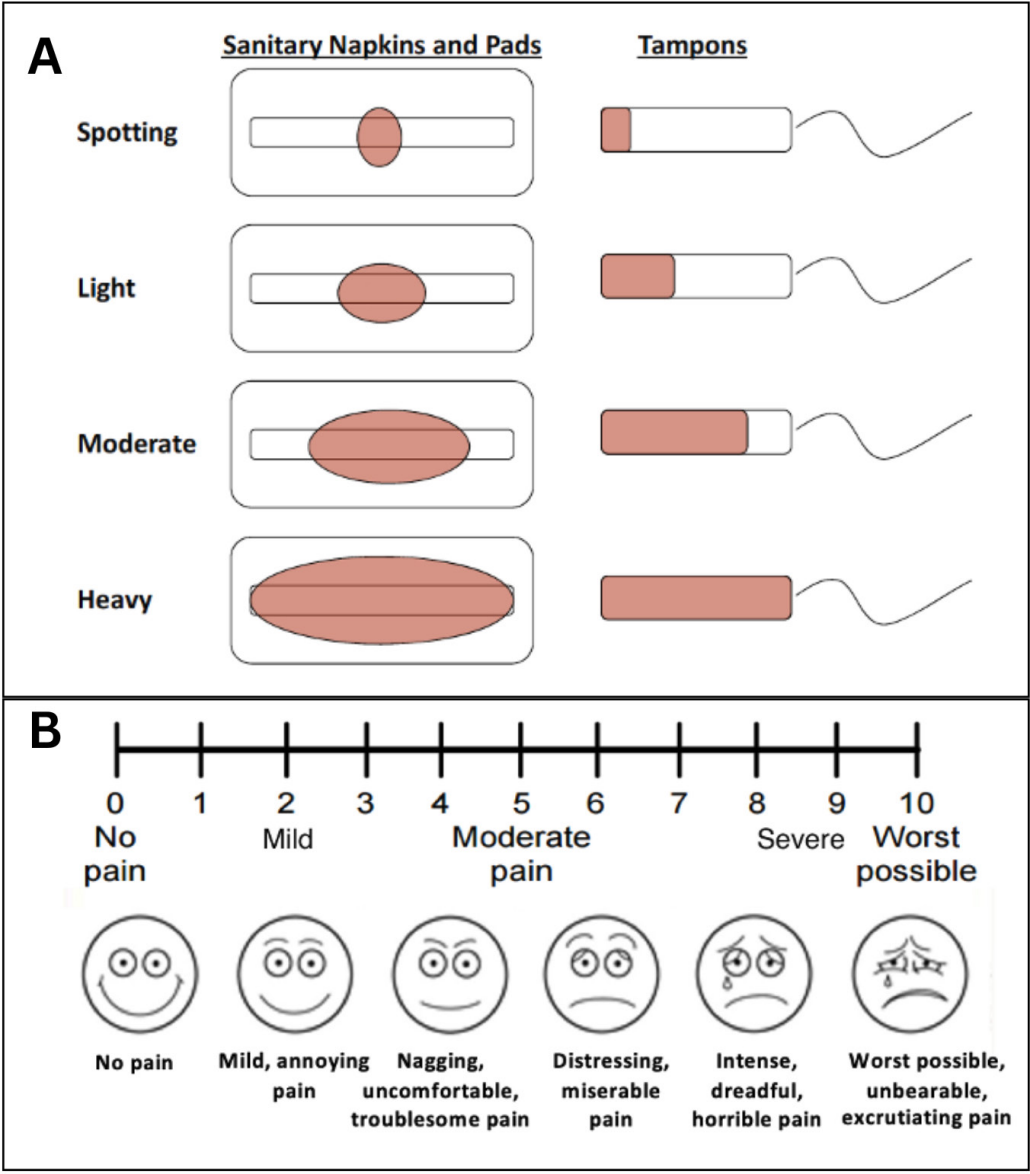
Pictographs used in the menstrual survey to aid respondents’ self-reported measurement of (A) bleeding heaviness and (B) pain level during menstruation.

- Dysmenorrhea (painful periods, >5).
- Age at menarche.
- Menstrual characteristics in the 3–6 months before pregnancy:
  - last menstrual period (LMP).
  - cycle regularity (how often and predictability within 1 week).
  - cycle frequency (days).
  - bleeding duration (days).
  - heaviness of flow (patient determined—see figure 2).
  - period pain levels (scale of 1–10—see figure 2).
  - premenstrual symptoms (eg, bloating, mood fluctuations, pelvic cramps).
- Contraception history: method(s) of contraception, duration of contraceptive use (years) and reason ceased.

#### Covariates

- Maternal age (years).
- Body Mass Index (BMI) at booking.
- Parity.
- Gravidity.
- Previous stillbirth.
- Previous preterm birth.
- Previous caesarean birth.
- Maternal self-reported country of birth.
- Smoking in pregnancy.
- Pre-existing maternal medical conditions (eg, diabetes, hypertension, thyroid disease).
- AUB treatment history (eg, hormonal or non-hormonal medication, iron supplementation or infusion, surgery).
- Maternal vaccinations.

### Data sources/ measurement

All data will be housed and linked in RedCap. All clinical, maternal, newborn and covariate data will be extracted from the woman’s electronic medical records. Data will be extracted from the pathology report for placentas sent to placental histopathology due to clinical indications. Participants’ self-reported exposure measures and fetal movements will be captured using specifically designed surveys. The menstrual history survey (online supplemental appendix 1) has been specifically developed using questions from the Menstrual Disorders of Teenagers (MDOT) Questionnaire,^41^ UCLA’s Department of Obstetrics and Gynaecology Patient History Questionnaire^42^ and the Growing Up Today Study Questionnaire.^43^ The survey is aided by pictographs for subjective measures such as pain level and heaviness of bleeding (figure 2). Women will complete this survey at the time of recruitment. A link to complete a fetal movements survey online (online supplemental appendix 2) will be electronically sent to women (via text message or email) at 28 and 36 weeks’ gestation.

### Potential bias and limitations

#### Selection bias and loss to follow-up

Women with menstrual abnormalities^44^ or who have had previous pregnancy complications may be more likely to participate when they hear the study’s aims. Selection bias will be mitigated by approaching all women at various antenatal clinics (different days and times). Similarly, we expect a degree of misclassification from those who do not report they have one of the excluded endometrial pathologies. We will cross-check with pre-existing medical records to mitigate this bias. Considering the latency of diagnosis of conditions such as endometriosis, it is likely that some participants will have underlying, undiagnosed endometrial pathologies. Endometriosis has an estimated prevalence of 10% in Australia,^45^ and pilot data from our research group has shown that 50% of women with endometriosis are not diagnosed until after pregnancy (unpublished data). This is anticipated but cannot be avoided. Our study will report the frequency of menstrual symptoms consistent with endometriosis in pregnant women for the first time. Our ethics also allow for the potential to recontact participants in the future and test the hypothesis that 5% of this cohort may go on to be diagnosed with endometriosis in the years after their pregnancy (beyond the scope of this protocol).

We will aim to recruit a diverse sample of participants. Given that menstrual characteristics may also differ by race or ethnicity,^46^ it is important to tease this out in this study. The study site has an extensive multicultural population, and research staff are accustomed to speaking with patients from multicultural backgrounds, including those from refugee communities. For non-English-speaking patients, the recruitment materials and survey will be translated into the top four languages (Dari, Vietnamese, Khmer, Mandarin) or can request interpreter services at the Health Service. Often, non-English-speaking patients attend their appointments with a family member (ie, a child or partner); as menstrual stigma prevails in various cultures,^47^ this may be an added barrier to discussing menstruation with these women. If less willingness to participate is observed, recruitment protocols will be revisited.

We anticipate loss to follow-up to be minimal as inclusion in the study requires women to be planning to receive all care and give birth at Monash Health. Where participants transfer care or give birth elsewhere, further participants will be recruited to maintain the sample size.

#### Measurement bias

There is the potential for recall bias regarding the menstrual and fetal movements survey. We have used validated menstrual history surveys to mitigate this. Evidence also suggests that most women recall the length of their menstrual cycle accurately within ±1 day, and reporting by sexually active women is more accurate.^48^ Given that this is a cohort of pregnant women, many of whom would have been trying to conceive, we expect recall of menstrual characteristics to have a high level of accuracy. Reports of heaviness and pain are more subjective, so pictograms and clinically used pain level scales have been incorporated into the survey to account for this (figure 2). With the uptake of menstrual cycle tracking applications,^49^ participants can also refer to this data if they have trouble with recall.

We anticipate that a higher proportion of respondents to the fetal movement survey will be primigravidae. Due either to greater interest in fetal movements because of the novel experience of late pregnancy for primigravidae or greater limitations on time among parous women. A national survey in New Zealand of fetal movements had greater participation by primigravidae (unpublished). Women in a first pregnancy are also more likely to present with decreased fetal movements and are more likely to experience some pregnancy complications, including pre-eclampsia and stillbirth.

Placentas from pregnancies complicated by the primary outcomes will be sent to pathology for investigation as per Health Service protocols. There is the potential that some placentas are missed or the family declines. We cannot mitigate this and will report the number of missing pathology reports to quantify this level of bias. Prospective consent to collect placental tissues may capture some of these outcomes. All other data will be obtained from medical records, minimising the risk of recall bias. The potential for missing or incorrectly entered data is minimal but cannot be avoided. Missing data will be coded as such to quantify this potential bias.

### Sample size

No previous research has compared the rates of stillbirth in women with and without abnormal menstrual characteristics. Previous estimates relating to pre-eclampsia^23 50^ found a 1.1-to-1.5-fold increased risk of stillbirth. Studies of stillbirth are notoriously difficult due to the requirement to recruit large numbers of women to be statistically powered. We have powered this study to be able to detect a 1.5-fold increase in a composite of stillbirth, spontaneous preterm birth, pre-eclampsia and FGR. Approximately 30% of women have AUB.^28 29^ The rate of the composite outcome at the study site is 14%. Therefore, assuming a 1.5-fold increased risk, 2:1 ratio, 80% power and two-tailed p<0.05, we will recruit 1104 women.

### Statistical analysis plan

A composite variable for AUB will be created based on ≥1 menstrual characteristic outside the normal range.^28^ The primary composite outcome will also be computed. All continuous data will be assessed for normality. Using standard statistical approaches, maternal and menstrual characteristics (individual and composite) will initially be compared between those who experienced the primary composite outcome and those who did not. Differences in menstrual characteristics will also be tabulated for individual components of the primary outcome. The associations between the AUB composite and individual components of the menstrual survey with the primary and secondary outcomes will then be determined using mixed-effects logistic regression, adjusting for potential confounders if appropriate. Principal component analysis will also be performed to identify which menstrual characteristics best predict the primary outcome. Area under the curve statistics will be used to assess model performance. Differences in fetal growth trajectory by exposure variables will also be determined using latent class trajectory models.^51^ Frequencies of placental pathology findings among women with AUB and the individual components of AUB will also be tabulated and compared. Planned secondary analyses will also examine the association between fetal movements, pregnancy outcomes and placental findings. Benjamani-Hochburg false discovery rate correction will be made to account for multiple testing. Statistical analyses will be conducted using Stata/SE 18.0 for Windows, and a two-tailed p<0.05 will be deemed statistically significant.

### Patient and public involvement

Participants were not asked or offered the opportunity to participate in the study design; however, the researchers have lived experience of menstruation, receiving or providing pregnancy care. The researchers considered the study requirements concerning participant convenience and comfort in answering menstrual questions.

### Ethics and dissemination

Ethical approval was obtained for this study on 24 April 2024 and will be conducted in accordance with the conditions of Monash Health Human Research Ethics Committee (MUHREC, Project ID: 83559). The confidentiality of all the participant’s data will be strictly maintained by all researchers in line with national and local guidelines. Future use of data will be in line with the ethics approval and if participants have consented to be recontacted. Before any future studies reusing data, a new ethics application or amendment would be made. Study outcomes will be disseminated at international conferences and published in peer-reviewed scientific journals. Lay reports will be made available to study participants on request after completion of the project.

## supplementary material

10.1136/bmjopen-2024-091813 online supplemental file 1

10.1136/bmjopen-2024-091813 online supplemental file 2
