# Supplementary material for: Your period and your pregnancy, a cohort study of pregnant patients investigating the associations between menstruation and birth outcomes in Australia: study protocol
Source: BMJ Open. 2025 Jan 22;15(1):e091813. doi: 10.1136/bmjopen-2024-091813 (PMC11784170; doi:10.1136/bmjopen-2024-091813)
Supplement: online supplemental file 1 [file bmjopen-15-1-s001.pdf]

Your Period and Your Pregnancy

Menstrual Survey

MENSTRUAL HISTORY:

This section asks about your menstrual experiences, characteristics and the symptoms you have experienced during your menstrual cycles. Please consider your average experience with your periods in the 3-6 months before becoming pregnant to answer these questions.

We understand that it may be difficult to recall all of this information; if you use a fertility or period tracking app, you can refer to it throughout to assist you. If not, please answer to the best of your ability.

1. At what age did you have your first period? _____ years old or ☐ Uncertain

2. When was your last period? *Known or approximate date:* ____ /____ /_______

3. Approximately how many periods do you have per year (prior to becoming pregnant)? _________

4. How long is your average menstrual cycle (i.e. how many days between the first day of menstrual bleeding between each period)? ______days or ☐ uncertain

5. How many days of bleeding would you usually have during your period? _____ days ☐ uncertain

6. Which best describes the regularity of most of your periods overall?

☐ Extremely regular (period starts 1-2 days before or after it is expected)

☐ Very regular (period starts 3-4 days before or after it is expected)

☐ Regular (period starts 5-7 days before or after it is expected)

☐ Somewhat irregular (period starts 8-20 days before or after it is expected)

☐ Irregular (period starts more than 20 days before or after it is expected)

☐ No pattern (period start times were inconsistent)

7. Do you experience spotting <u>before</u> a period?

☐ Never

☐ Rarely

☐ Often

☐ Almost Always

8. Do you experience bleeding between your expected periods (intermenstrual bleeding)? i.e. more than spotting – requires use of a sanitary product

☐ Never

☐ Rarely

☐ Often

☐ Almost Always

9. Based on the diagram below, please tick the average amount of bleeding you experience every four hours during your period at its heaviest:

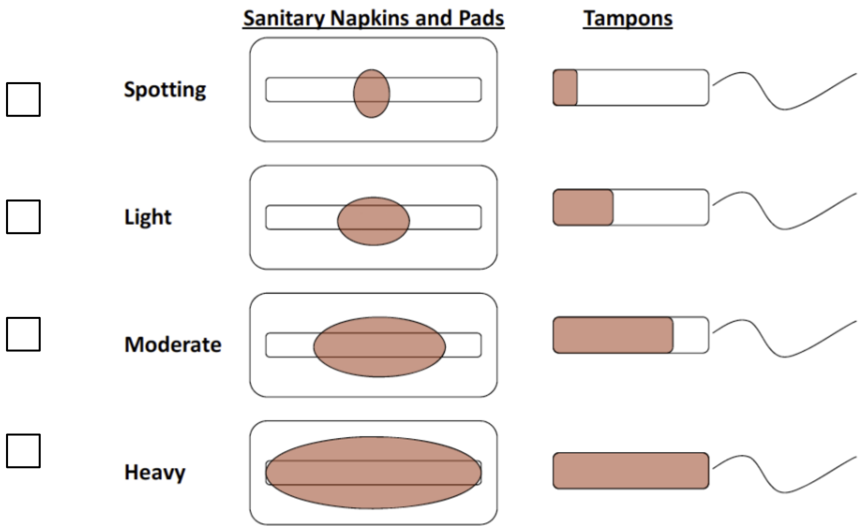

10. Based on the diagram below, on a scale of 0-10 please circle the average amount of period pain you experience during your periods?

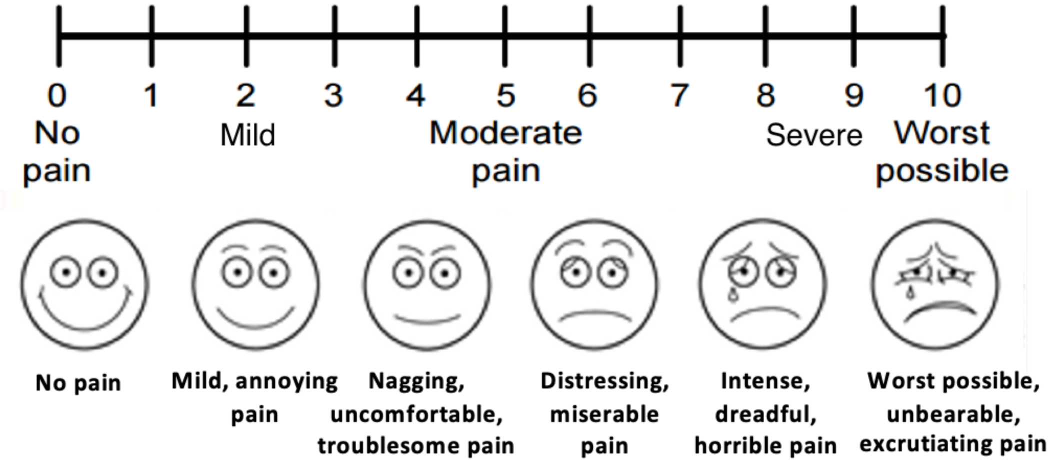

11. Do you regularly use anything to relieve symptoms for period pain?

☐ Medication (*please specify*): ____

☐ Heat Pack/Hot Water Bottle

☐ Nothing

☐ Other ____

12. If you ticked yes to regularly taking medication for period pain, on a scale of 0-10 (*with zero being not effective at all and 10 being highly effective*), how effective was it in relieving your pain? _________

13. Do you regularly experience any of the following? (*Please tick all that apply)*

☐ Flooding through to clothes or bedding

☐ Frequent change of sanitary pads or tampons (changing every 2 hours or less)

☐ Requiring double sanitary protection (i.e pad and tampon)

☐ Pass large blood clots

☐ Spotting throughout the cycle

14. Do you regularly experience any form of the following sexual dysfunctions?

☐ Dyspareunia (persistent or recurrent genital or pelvic pain during or after sexual intercourse)

☐ Vaginismus (painful tightening/contractions of the vagina during penetration)

☐ Low libido

☐ Post-coital bleeding (after sex)

☐ Other: _______________

15. Thinking about your periods more generally, have you ever had an iron infusion or regularly take iron tablets due to menstruation? *Please tick all that apply*

☐ Yes – iron infusion

☐ Yes – iron tablets

☐ No

16. Thinking about your periods more generally, have you ever seen a health professional (including alternative/complementary therapies) in relation to your period or undergone medical investigations related to your period?

e.g. acupuncture/blood tests/scans

Please circle Yes / No *and detail below:*

____

____

____

____

____

____

____

17. In relation to your periods overall, do you commonly experience any of the following? *Tick all that apply*

		No/ never	Just before a period	At the time of period	Any time of month	All the time	Some-times	Don't know
Symptoms:								
Nausea								
Vomiting								
Bloating								
Diarrhoea								
Constipation								
Indigestion, reflux, heartburn								
Changes in appetite								
Aching around the vagina								
Breast tenderness								
Pelvic pain	*Aching*							
	Cramping							
	Stabbing							
	Other (please specify)							
Lower back pain								
Pain during or after urination								
Pain when bladder is full								
Need to pass urine often								
Pain when passing wind								
Pain when emptying bowels								
Urgent need to empty bowels								
Bleeding from anus								
Thrush (itchy or sore vagina)								
Feeling fatigued								
Insomnia								
Headaches								
Dizziness or fainting								
Feeling depressed								
Irritability/mood swings								

18. Thinking about your periods more generally, how do your periods affect your lifestyle? (*Please circle a number between 0-10)*

0 = no interference, 10 = major interference, N/A = not applicable to me

**Activity**	**Scale of 0 to 10**											**N/A**
Education/training	0	1	2	3	4	5	6	7	8	9	10	
Attending work	0	1	2	3	4	5	6	7	8	9	10	
Home duties	0	1	2	3	4	5	6	7	8	9	10	
Social activities	0	1	2	3	4	5	6	7	8	9	10	
Relationship with family	0	1	2	3	4	5	6	7	8	9	10	
Relationship with friends	0	1	2	3	4	5	6	7	8	9	10	
Relationship with partner	0	1	2	3	4	5	6	7	8	9	10	
Sexual activity	0	1	2	3	4	5	6	7	8	9	10	
Sport and exercise	0	1	2	3	4	5	6	7	8	9	10	

19. What was it about your period that affects your life?

0 = no interference, 10 = major interference, N/A = not applicable to me

**Symptom**	**Scale of 0 to 10**											**N/A**
Pain	0	1	2	3	4	5	6	7	8	9	10	
Heavy blood flow	0	1	2	3	4	5	6	7	8	9	10	
Tiredness/fatigue	0	1	2	3	4	5	6	7	8	9	10	
Moods	0	1	2	3	4	5	6	7	8	9	10	
Generally feeling unwell	0	1	2	3	4	5	6	7	8	9	10	
Other _________________	0	1	2	3	4	5	6	7	8	9	10	

20. Does the above interference occur with?

☐ Some periods

☐ Most periods

☐ All periods

☐ N/A

CONTRACEPTION HISTORY

21. Please detail your contraceptive history prior to becoming pregnant, as far back as you can remember.

Please estimate the dates to the best of your ability (month and year).

Examples of contraceptive methods are provided below:

1. Condom
2. Diaphragm
3. Oral Contraceptive Pill (OCP)
4. Depo-Provera Injection
5. Hormonal Intrauterine Device (IUD) *(i.e. Mirena)*
6. Copper IUD
7. Implant *(i.e. Implanon/Rod)*
8. Withdrawal
9. Abstinence
10. Emergency Contraception Pill
11. Hormonal Ring
12. Sponge
13. Contraceptive Patch
14. Cervical Cap
15. Spermicide
16. Other

Contraceptive Used	Month/Year started	Month/Year ended	Reason Ceased
e.g. 7. Implanon	*2012*	*2013*	*Side-effects or trying to conceive*

MEDICAL HISTORY

22. Have you ever been diagnosed with any of the following reproductive conditions in the past?

Please tick all that apply:

- ☐ Polycystic Ovarian Syndrome (PCOS)
- ☐ Fibroids
- ☐ Polyps
- ☐ Pelvic Inflammatory Disease (PID)
- ☐ Adenomyosis
- ☐ Asherman's Syndrome
- ☐ Blocked tubes
- ☐ Heavy Menstrual Bleeding (HMB) or Abnormal Uterine Bleeding (AUB)
- ☐ Irregular ovulation
- ☐ Retroverted uterus
- ☐ Chlamydia/Gonorrhoea
- ☐ Other ____
- ☐ None

Your story:

We understand that the everyone's experiences will be different and cannot always be captured in a questionnaire. Is there anything else you would like to add that you don't think was captured in this survey regarding your menstrual history that may be of interest?

____

____

____

____

____

____

____

____
