# Supplementary material for: Your period and your pregnancy, a cohort study of pregnant patients investigating the associations between menstruation and birth outcomes in Australia: study protocol
Source: BMJ Open. 2025 Jan 22;15(1):e091813. doi: 10.1136/bmjopen-2024-091813 (PMC11784170; doi:10.1136/bmjopen-2024-091813)
Supplement: online supplemental file 2 [file bmjopen-15-1-s002.pdf]

Your Period and Your Pregnancy

Fetal Movements Survey

The following questions relate to your baby's movements. Every mother and baby are different and there are no right or wrong answers.

1: In this pregnancy, are you able to feel baby's movements?

01 Yes (If yes, go to Question 2)

02 No/Not yet

(If 'no' or 'not yet' survey ends with message 'Thank you for your response. Every mother and baby are different and some feel movements earlier or later in pregnancy than others. If you are concerned about fetal movements contact your care provider')

2. How easy do you find it to feel baby movements?

(Please select one answer only)

01 Very easy to feel

02 Somewhat easy to feel

03 Somewhat difficult to feel

04 Very difficult to feel

3. In the last two weeks did the <u>strength</u> of your baby's movements

(Please select one answer only)

01 Increase

02 Decrease

03 Stay the same

4. During the last two weeks did the <u>frequency</u> of your baby's movements

(Please select one answer only)

01 Increase

02 Decrease

03 Stay the same

5. During the last two weeks were your baby's movements

(Please select one answer only)

01 Always strong

02 Always soft

03 Both strong and soft

6. Busy Times are when a baby is active or 'awake' and there is not just one movement but many over a period of time from 15 minutes to an hour or more.

In the last week, you felt your baby have busy times:

(Please select one answer only)

01 No busy times felt in the last week – *If this answer is selected, skip Q7*

02 Once in the last week

03 More than once in the last week but not every day

04 Once every day

05 More than once every day

7. In the last week on average the length of these 'busy times' were usually:

(Please select one answer only)

01 No busy times in the last week

02 Longer than before

03 The same as before

04 Shorter than before

8. Overall, how would you describe your baby's activity level?

(Please select one answer only)

01 Extremely active

02 Moderately active

03 Somewhat active

03 Not very active

9. Hiccups feel like your baby doing small rhythmic jerking movements that can carry on for a several minutes.

In the last week, you have felt your baby having hiccups:

(Please select one answer only)

01 No hiccups felt in the last week

02 Once in the last week

03 More than once in the last week but not every day

04 Once every day

05 More than once every day

10. In the last week, in the mornings (6am to midday) your baby's movements were:

01 Strong

02 Moderate

03 Soft/quiet

11. In the last week, in the afternoons (midday to 6pm) your baby's movements were:

01 Strong

02 Moderate

03 Soft/quiet

12. In the last week, in the evenings (6pm to midnight) your baby's movements were:

01 Strong

02 Moderate

03 Soft/quiet

13. In the last week, overnight (midnight to 6am) your baby's movements were:

01 Strong

02 Moderate

03 Soft/quiet
