# Supplementary material for: Your period and your pregnancy, a cohort study of pregnant patients investigating the associations between menstruation and birth outcomes in Australia: study protocol
Source: BMJ Open. 2025 Jan 22;15(1):e091813. doi: 10.1136/bmjopen-2024-091813 (PMC11784170; doi:10.1136/bmjopen-2024-091813)
Supplement: Supplementary file 3 [file bmjopen-2024-091813.reviewer_comments.pdf]

PEER REVIEW HISTORY

BMJ Open publishes all reviews undertaken for accepted manuscripts. Reviewers are asked to complete a checklist review form and are provided with free text boxes to elaborate on their assessment. These free text comments are reproduced below.

ARTICLE DETAILS

Title (Provisional)

Your Period and Your Pregnancy, a Cohort Study of Pregnant Patients Investigating the Associations Between Menstruation and Birth Outcomes in Australia: Study Protocol

Authors

Tindal, Kirstin; Cousins, Fiona; Palmer, Kirsten Rebecca; Ellery, Stacey; Vollenhoven, Beverley; Gargett, Caroline E; Gordon, Adrienne; Bradford, Billie; Davies-Tuck, Miranda

---

VERSION 1 - REVIEW

Reviewer 1

Name Tunç, Mehmet

Affiliation Division of Reproductive Endocrinology and Infertility, Department of Obstetrics and Gynecology, Başkent University Hospital

Date 17-Sep-2024

COI None

I believe that your study will provide a significant contribution to the literature.

Reviewer 2

Name Yang, Yihua

Affiliation Guangxi Traditional Chinese Medical University Affiliated First Hospital

Date 24-Dec-2024

COI I declare that I have no competing interests in relation to the manuscript I am reviewing. Additionally, there are no conflicts of interest that could influence my objective evaluation of the work.

AUB treatment history may influence study results.As treatment may alter AUB symptoms, affecting the association between menstrual characteristics and pregnancy outcomes.The

study protocol does not mention AUB treatment history.The authors are recommended to collect AUB treatment history data and consider its impact in the data analysis.

VERSION 1 - AUTHOR RESPONSE

*Reviewer 2: AUB treatment history may influence study results. As treatment may alter AUB symptoms, affecting the association between menstrual characteristics and pregnancy outcomes. The study protocol does not mention AUB treatment history. The authors are recommended to collect AUB treatment history data and consider its impact in the data analysis.

We have added "AUB treatment (e.g. hormonal or non-hormonal medication, iron supplementation or infusion, surgery)" to the covariates collected in this study – pg7.

We thank reviewer 2 for their comment.
